# Efficacy and safety of low-dose naltrexone (LDN) in fibromyalgia: a systematic review and meta-analysis

**DOI:** 10.1097/MS9.0000000000003203

**Published:** 2025-03-28

**Authors:** Muhammad Hashir Nazir, Uswa Mehboob, Muhammad Farhan, Tirath Patel, Muhammad Ahmad, Saleha Nazir, Tooba Ahmed Durrani, Mustafa Khafaja, Abdulaziz Sobhi, Mariyam Kuznetsova, Muhammad Ahmed, Ayoola Awosika

**Affiliations:** a Department of Internal Medicine, King Edward Medical University, Lahore, Pakistan; b Department of Internal Medicine, College of Medicine, Ajman University, Ajman, UAE; c Department of Internal Medicine, Trinity Medical Sciences University School of Medicine, Ratho Mill Kingstown, Saint Vincent and Grenadines; d Department of Internal Medicine, Dubai Medical University, Dubai, UAE; e Department of Internal Medicine, Henry Ford Macomb Hospital, Clinton Township, Michigan; f Department of Clinical Medicine, University of Illinois College of Medicine Peoria, Peoria, Illinois

**Keywords:** fibromyalgia, LDN, low-dose naltrexone, naltrexone

## Abstract

**Background:**

Fibromyalgia is a chronic disorder characterized by pain and psychological symptoms in adults. Several randomized controlled trials (RCTs) have shown the effectiveness and safety of low-dose naltrexone (LDN) in the treatment of fibromyalgia in variable small settings. Hence the need to conducted a meta-analysis to evaluate the overall effect and the strength of evidence.

**Methodology:**

PUBMED, CENTRAL, and ClinicalTrials.gov were searched to retrieve RCTs comparing naltrexone with placebo in fibromyalgia patients for systematic review and meta-analysis. We conducted pairwise meta-analyses using DerSimonian and Laird random-effects model via RevMan 5.4. We reported dichotomous outcomes as relative risk (RR) and continuous outcomes as standardized mean difference (SMD) with 95% confidence intervals (CIs). Quality of included RCTs was assessed using revised Cochrane Risk of Bias Tool for RCTs (RoB 2.0). Heterogeneity was detected by Chi^2^ and *I*^2^ values for each meta-analysis and if significant, sensitivity analysis was performed.

**Results:**

We included five RCTs in meta-analysis. Our results estimated that LDN is superior to placebo in alleviating pain both in primary (SMD −0.61; 95% CI −1.14, −0.08) and sensitivity analysis (SMD −0.87; 95% CI −1.28, −0.46) but not in raising mechanical pain threshold (SMD 0.24; 95% CI −0.09, 0.56) in fibromyalgia patients. Neither the primary (RR 1.68; 95% CI 0.84, 3.36) nor sensitivity analysis (RR 0.98; 95% CI 0.72, 1.34) could associate LDN use with the incidence of headache. The incidence of vivid dreams (RR 2.41; 95% CI 1.77, 3.28) was significantly higher in treatment group as compared to placebo.

**Conclusion:**

LDN is considered to be effective in the treatment of fibromyalgia. No serious adverse effects were reported in treatment group. There is need for more RCTs to support the evidence.

## Introduction

Fibromyalgia is a complex chronic musculoskeletal hyperalgesic syndrome characterized by complicated set of unrelated symptoms of variable severity which include musculoskeletal pain, physical and mental fatigue, sleep disturbances, parasthesia, memory and concentration problems, gastrointestinal symptoms, headaches, depression, and functional symptoms. Patients usually report chronic widespread pain similar to neuropathic pain along with development of “tender points” due to abnormal pain processing^[1,2]^. Fibromyalgia is common in general population having prevalence between 0.2% and 6.6% with a high prevalence of 6.4% in the USA and an incidence of 4.3 per 1000 persons years. The disease is most common in middle-aged females with existent rheumatic diseases^[3,4]^. Other risk factors for fibromyalgia are obesity, smoking, alcohol abstinence and preexisting disorders such as diabetes, irritable bowel syndrome, patients undergoing hemodialysis and systemic lupus erythematosus^[4,5]^.

**HIGHLIGHTS**

- Low-dose naltrexone (LDN) significantly reduces pain severity in fibromyalgia patients.
- LDN is well-tolerated but increases vivid dreams vs placebo.
- LDN reduces pain intensity but has limited impact on pain thresholds.
- This is the first meta-analysis of LDN for fibromyalgia.
- Larger, high-quality randomized controlled trials are needed to validate LDN’s efficacy.

According to the 2010/2011 fibromyalgia diagnostic criteria of the American College of Rheumatology (ACR), fibromyalgia is diagnosed in adults when generalized pain in at least 4 of 5 regions is present for at least 3 months, widespread pain index (WPI) >7 and symptom severity scale (SSS) score ≥5 or WPI of 4–6 and SSS score ≥9^[6]^. The newly developed AAPT core diagnostic criteria for fibromyalgia requires multisite pain (MSP) at ≥6 sites out of 9 in conjunction with moderate to severe sleep disturbance and/or physical or mental fatigue that have been present for 3 months in the presence or absence of supportive symptoms such as tenderness, dyscognition, musculoskeletal stiffness and central sensitivity to light and noise, and related disorders^[7]^. Treatment of fibromyalgia constitutes non-pharmocological arm including patient education on management of anxiety and major depression using stress reduction and relaxation techniques and maintenance of sleep hygiene^[2]^ cognitive behavioral therapy^[8]^ and cardiovascular exercises such as aquatic^[9]^, aerobic and resistance training exercises^[10]^ and pharmacological arm including selective serotonin reuptake inhibitors (SSRIs), serotonin and norepinephrine reuptake inhibitors (SNRIs)^[11]^ and tricyclic antidepressants (TCAs)^[12]^ or combination of these^[13]^.

Low-dose naltrexone (LDN), a recently employed treatment for fibromyalgia due to its ability to reduce glial inflammatory response and upregulate opioid signaling by regulating Toll-like receptor 4 (TLR-4) and transient opioid receptor blockade. Despite its divergence from traditional antagonistic effect at standard dose^[14]^, it has been shown to be effective and safe in various randomized controlled trials (RCTs) and cohort studies in fibromyalgia patients^[15]^. The evidence of LDN efficacy and safety in fibromyalgia patients is limited to few systematic reviews which have argued the lower certainty of evidence due to variability or lack of data in comprehensive narrative analysis^[16,17]^. However, no meta-analysis has been conducted to date to statistically establish the safety and superiority of LDN pharmacotherapy in fibromyalgia patients.

Therefore, we aimed to conduct the first meta-analysis of available RCTs on effectiveness and safety of LDN in fibromyalgia patients to fill this knowledge gap. Our article is thus directed to systematically review as well as statistically investigate the efficacy and safety of LDN in fibromyalgia and ultimately evaluating its usefulness in clinical practice.

## Materials and methods

### Study design

This meta-analysis was adapted to The Cochrane Handbook for Systematic Reviews of Interventions recommendations and reported in accordance with the Preferred Reporting Items for Systematic Reviews and Meta-Analysis (PRISMA) statement^[18,19]^. Because the study was a meta-analysis, no ethical approval was required. The protocol of the study was preregistered with Open Science Framework (OSF)^[20]^ and International Prospective Register of Systematic Reviews (PROSPERO) under identifier CRD42024587800.

### Data sources and searches

We searched PUBMED, Cochrane Central Register of Controlled Trials (CENTRAL), and ClinicalTrials.gov for abstracts and articles eligible for this meta-analysis. We also manually screened reference lists of included studies and relevant systematic reviews to extract RCTs compliant with our inclusion criteria. Neither diagnostic or ethnographic restrictions nor time limit were applied for publications during screening.

### Eligibility criteria

The inclusion and exclusion criteria for the selection of studies in the systematic review are outlined in (Table 1).

**Table 1:** Inclusion and exclusion criteria.

Inclusion criteria	Exclusion criteria
Study design: Randomized controlled trials (RCT)	Studies other than RCTs
Population: Patients suffering from fibromyalgia	Studies with comparator other than placebo
Intervention: Low-dose naltrexone	Studies in languages other than English
Comparator: Placebo	Closed access articles
Studies available in English language	
Open access articles	

### Screening and data extraction

We searched databases for relevant RCTs using keywords like *fibromyalgia, pain in fibromyalgia, chronic pain disorders, LDN, Low-dose naltrexone, naltrexone*. After deduplication, two authors (U.M. and M.H.N.) worked independently on title and abstract screening followed by full text screening to identify eligible RCTs and exclude the rest. Any disagreements in the selection procedure were resolved by discussion and involvement of third author (M. Ahmad), if needed. The complete selection process is summarized in a PRISMA flow chart.

A single author (S.N.) then extracted the information regarding study design and publication time, population characteristics, intervention dose, follow-up duration, and outcome measures from included studies into Microsoft Excel 2019. The extracted data was then reviewed independently by two authors (M.F. and T.A.D.) and contradictions were settled by discussion.

### Outcome measures

Our primary outcome measure was pain severity. The secondary outcomes of interest were mechanical pain threshold, incidence of headache, and incidence of vivid dreams.

### Risk of bias assessment

Two authors (M. Khafaja and A.S.) independently assessed the quality and risk of bias in the included papers using the revised Cochrane Risk of Bias Tool for Randomized Controlled Trials (RoB 2.0)^[21]^. Following five domains are addressed by ROB 2.0: (1) bias arising from the randomization process; (2) bias due to deviations from intended interventions; (3) bias due to missing outcome data; (4) bias in the measurement of the outcome and (5) bias in the selection of the reported result. The disagreements in the assessment of bias were settled by consensus and involvement of other authors.

### Data analysis

We performed meta-analyses using Review Manager, Version 5.4. Outcome measures were statistically analyzed using DerSimonian and Laird random-effects model. We reported dichotomous outcomes as relative risk (RR) along with 95% confidence interval (CI). The effect sizes for continuous outcomes were calculated as standardized mean difference (SMD) with 95% CI. For each continuous outcome, we calculated the effect size as the difference between post-intervention and baseline means in both the intervention and control groups. The missing correlation coefficient values for estimation of standard deviation for change were imputed in accordance with *Cochrane Handbook for Systematic Reviews of Interventions* from the source within the meta-analysis that reported baseline and final values of standard deviation as well as standard deviation for change. Otherwise, correlation coefficient was conservatively set at 0.5 for the included studies in which the source within meta-analysis or any excluded relevant study did not report standard deviation for change or correlation coefficient for the outcome of interest^[19,22,23]^. For the studies which reported CIs for the change in mean value, standard deviation for change was imputed from the CI.

Heterogeneity was assessed using Chi^2^ and *I*^2^ statistics for each meta-analysis. For Chi^2^ test, *P* < 0.1 was considered significant while *I*^2^ > 50% was considered substantial^[24,25]^.

### Subgroup and sensitivity analyses

We planned to conduct subgroup analyses on primary and secondary outcomes with significant heterogeneity (Chi^2^ *P* < 0.1, *I*^2^ > 50%) on the basis of trial design and intervention dosage. We also conducted sensitivity analyses on primary and secondary outcomes with substantial heterogeneity (Chi^2^ *P* < 0.1, *I*^2^ > 50%) with number of studies >2.

### Changes from protocol

#### Data sources

A trial register ClinicalTrials.gov was additionally searched.

#### Eligibility criteria

The criterion for patient age is no longer required in the present study due to lack of data.

#### Secondary outcomes

Sleep quality, fatigue, QoL scores, and mood elevation were replaced by mechanical pain threshold in accordance with available data and Safety outcomes (incidence of headache and vivid dreams) were added.

## Results

### Screening process

**Figure 1.**
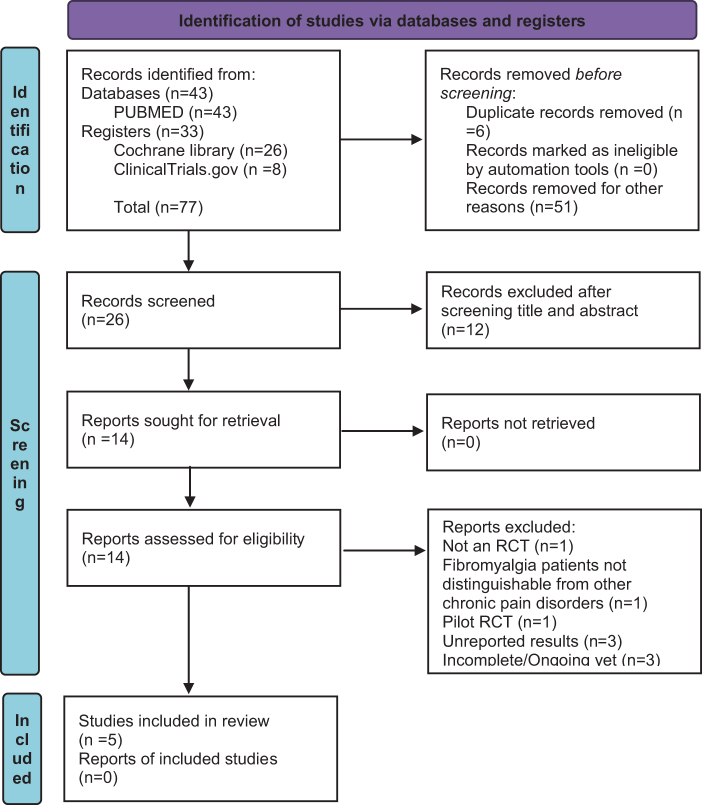
PRISMA 2020 flowchart: Trial flow of retrieval and inclusion of eligible studies.

The screening process identified 77 records, out of which we retrieved and assessed 14 articles for eligibility. Only five of these articles met the inclusion criteria and were included in review and meta-analysis^[26–30]^. In order to analyze the maximum useable data, both the abstracts and full publications were included in the present study, if they met the inclusion criteria. The entire selection and screening process is summarized in Figure 1.

### Characteristics of included studies

Out of the five included studies, two were conducted in Denmark, two in the USA and one in Egypt. While three of the studies used the ACR 1990 diagnostic criteria for fibromyalgia^[31]^ as diagnostic criteria, one used ACR 2011 and the fifth relied on physician’s diagnosis confirmed by evaluation during first study visit. All of the included studies are double-blind in design. Most of the RCTs covered female patients only while many patients were taking concomitant medication. Among the highly variable outcomes studied by RCTs, pain intensity, pain threshold and Fibromyalgia Impact Questionnaire (revised) were among the most evaluated outcomes. The characteristics of included RCTs and the characteristics of patients are summarized in Supplemental Digital Content Table 1, available at: http://links.lww.com/MS9/A776.

### Risk of bias in included studies

**Figure 2.**
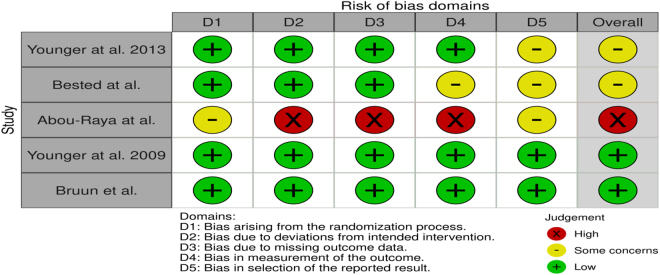
Risk of bias in included studies.

Among the included studies, 40% RCTs had some concerns for bias due to moderate risk of bias in one or multiple domains including measurement and reporting of outcomes. One of the RCTs, abstract type, had high risk of bias primarily due to lack of detailed methods and outcome measurements. Remaining two RCTs had low risk of bias in all domains. ROB 2.0 assessment of included RCTs is demonstrated in (Fig. 2).

### Quantitative data synthesis

#### Primary outcome

##### Pain severity

**Figure 3.**
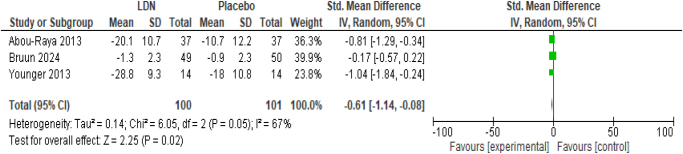
Forest plot of comparison: (1) LDN vs placebo, outcome: (1.1) change in pain intensity.

The outcome was measured as the change from baseline pain severity on visual-analogue scale or Numerical Rating Scale (NRS). Significant reduction in pain severity was observed with LDN as compared to placebo (SMD −0.61; 95% CI −1.14, −0.08; *P* = 0.02) (Fig. 3). Substantial heterogeneity was detected among the studies (*I*^2^ = 67%).

#### Secondary outcomes

##### Mechanical pain threshold

**Figure 4.**
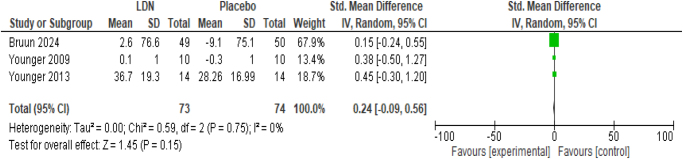
Forest plot of comparison: (1) LDN vs placebo, outcome: (1.2) change in mechanical pressure threshold.

The outcome was measured as the change in mechanical pain threshold between baseline and post-intervention levels. LDN intervention could not raise the mechanical pain threshold significantly in fibromyalgia patients as compared to placebo (SMD 0.24; 95% CI −0.09, 0.56; *P* = 0.15) (Fig. 4). The meta-analysis did not report any heterogeneity among the studies (*I*^2^ = 0%).

##### Incidence of headache

**Figure 5.**
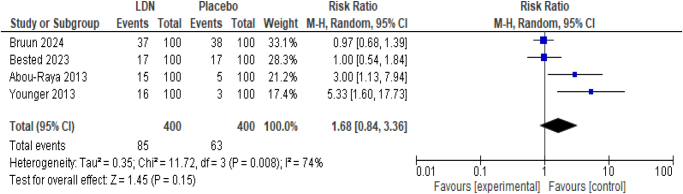
Forest plot of comparison: (1) LDN vs placebo, outcome: (1.3) Incidence of headache.

LDN intervention did not significantly cause the incidence of headache in fibromyalgia patients as compared to placebo (RR 1.68; 95% CI 0.84, 3.36, *P* = 0.15) (Fig. 5). However, significant heterogeneity was detected among the studies (*I*^2^ = 74%).

##### Incidence of vivid dreams

**Figure 6.**
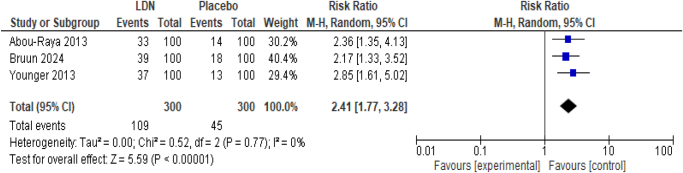
Forest plot of comparison: (1) LDN vs placebo, outcome: (1.4) incidence of vivid dreams.

LDN intervention was significantly associated with the incidence of vivid dreams as compared to placebo in fibromyalgia patients (RR 2.41; 95% CI 1.77, 3.28, *P* < 0.00001) (Fig. 6). There was no heterogeneity among the studies (*I*^2^ = 0%).

#### Subgroup analysis

We could not perform our prior defined subgroup analyses due to lesser number of studies, lack of useable data, or similar trial design/conditions in the included RCTs.

#### Sensitivity analyses

We performed sensitivity analysis for change in pain intensity and incidence of headache which are the only outcomes with significant heterogeneity.

##### Change in pain intensity

**Figure 7.**
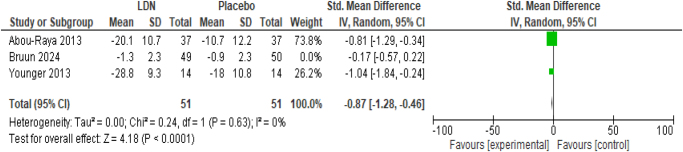
Sensitivity analysis of change in pain intensity.

The exclusion of the only study with NRS scale of pain intensity not only enhanced the significance of effect (SMD −0.87; 95% CI −1.28, −0.46; *P* < 0.0001) but also removed the heterogeneity among the studies (*I*^2^ = 0%) (Fig. 7).

##### Incidence of headache

**Figure 8.**
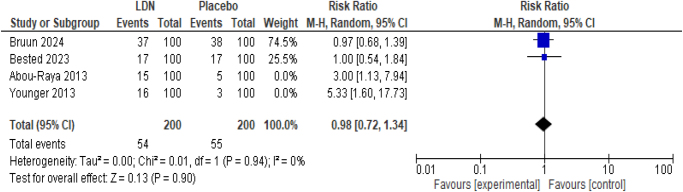
Sensitivity analysis of incidence of headache.

The exclusion of study/studies with some concern of bias in multiple domains did not alter the significance of effect (RR 0.98; 95% CI 0.72, 1.34, *P* = 0.90) but removed the heterogeneity among studies (*I*^2^ = 0%) (Fig. 8).

#### Publication bias

Funnel plots could not be constructed due to number of studies <10 as the power of tests is insufficient to discriminate chance from real asymmetry as per *The Cochrane Handbook for Systematic Reviews of Interventions*.

## Discussion

Based on this study, the use of LDN demonstrated significant reduction in pain severity with no raise in mechanical pain threshold as compared to placebo in fibromyalgia patients. LDN was not found to be associated with incidence of headache in patients. LDN group, however, experienced significantly higher incidence of vivid dreams than placebo group. The chance of error and controversial strength is present due to small sample size and heterogeneity.

Our results are not concordant with the findings of other meta-analyses employing LDN in chronic pain and inflammatory disorders but the quality of their evidence may have been suboptimal^[32]^. This may be explained by an entirely different morbidity and extremely small number of included studies in previous studies. Several cohort studies have, however, established superiority of LDN in fibromyalgia patients which is consistent with our meta-analysis as well as individual RCT results^[15]^. Regarding safety of LDN, no meta-analyses reported any significant association of serious adverse events with LDN intervention however, incidence of some minor adverse events, different from our study, was found significantly higher in the intervention group than control^[33]^.

Recent evidence describes fibromyalgia as a potential inflammatory disorder in which cyclic neuronal and inflammatory cell interaction results in elevated cytokines and neuropeptide levels which have been correlated with elevated pain perception and sensitization in fibromyalgia patients^[34]^. LDN has been demonstrated to block TLR-4 in macrophages and microglia, inhibit or reduce T-cell proliferation and increase phagocytic activity of macrophages by raising IL-2 and TNF-α concentration and is therefore considered effective in chronic inflammatory disorders like fibromyalgia^[35]^. This mechanism of action explains LDN role as analgesic, anti-inflammatory, and immunomodulator suggesting its efficacy in similar chronic pain, inflammatory and fatigue disorders such as multiple sclerosis and chronic fatigue syndrome thus enhancing advantageous role of LDN in the treatment of comorbidities associated with fibromyalgia, chronic fatigue and even cancer^[7,36–38]^. The observed benefits of LDN in fibromyalgia patients may be attributed to its immunomodulatory properties, which align with mechanisms proposed in other emerging therapies. Notably, stem cell therapy has demonstrated efficacy in neurological disorders through immune response modulation and tissue repair^[39]^. These parallels suggest a potential avenue for further research into the shared pathways of these treatments.

Despite some consensus on LDN efficacy in fibromyalgia, varying dosing regimens of LDN from as low as 0.1 mg to as high as 9–50 mg once or twice daily, have been adopted throughout literature^[40]^. In a single-blinded clinical trial, Bruun- Bruun-Plesner *et al*^[41]^ estimated the effect of test doses between 0.75–6 mg on 10 fibromyalgia symptoms in 25 patients. Their calculations found ED50 to be 3.88 mg and ED95 to be 5.40 mg in sample and their dose-response analysis established 4.5 mg to be the most feasible and efficacious dose for fibromyalgia patients^[41]^. Many patients included in this meta-analysis were taking concomitant medication which has been shown to have insignificant interaction with LDN intervention^[15]^. Naltrexone has been employed in the treatment of alcohol-use disorders despite unobvious efficacy^[42]^ and while 1 of the included RCTs^[30]^ excluded alcohol abusers, rest of the RCTs neither excluded nor mentioned/considered confounding effect of alcohol abuse.

This is the first meta-analysis that has compiled and analyzed all the RCTs, both abstracts and full publications (except pilot RCTs), which reported placebo-controlled efficacy and safety results of LDN in fibromyalgia patients. Although several systematic reviews have performed comprehensive narrative analysis previously, no statistical meta-analysis of LDN efficacy and safety has been published to date. But substantial heterogeneity among the included studies, moderate to high risk of bias and lesser number of studies with smaller sample sizes limit the quality and certainty of evidence. The heterogeneity in the studies arose due to smaller size and variability in results while some heterogeneity is inevitable due to clinical and methodological differences. Since funnel plots could not be constructed due to lesser number of studies, Chi^2^ statistic was used to determine heterogeneity which may not be reliable and therefore sensitivity analyses were performed to reduce heterogeneity^[43,44]^. The unaltered significance of effect with sensitivity analyses has, however, improved the quality and certainty of evidence.

Although LDN efficacy in fibromyalgia is emphasized by various RCTs and cohort studies, our meta-analysis is in agreement with some of the evidence but has also highlighted the controversy in some results of previous studies which indicates the need for further trials and analyses in this regard.

## Conclusion

The present meta-analysis concluded that there is improvement in pain severity but not in sensation with LDN use in fibromyalgia patients. LDN is not found to cause any major adverse events but has considerable association with minor adverse events like vivid dreams. There may be some controversy in evidence due to small sample size and moderate to high risk of bias but sensitivity analyses have enhanced quality of evidence. There is a need to conduct more RCTs with lower risk of bias and larger sample size.

## Data Availability

All the relevant data have been added in the manuscript and is available from corresponding authors on request.

## Research registration unique identifying number (UIN)

Our study was registered at PROSPERO under registration ID CRD42024587800 https://www.crd.york.ac.uk/PROSPERO/.
